# Confocal laser scanning, scanning electron, and transmission electron microscopy investigation of *Enterococcus faecalis* biofilm degradation using passive and active sodium hypochlorite irrigation within a simulated root canal model

**DOI:** 10.1002/mbo3.455

**Published:** 2017-02-28

**Authors:** Saifalarab A. Mohmmed, Morgana E. Vianna, Matthew R. Penny, Stephen T. Hilton, Nicola Mordan, Jonathan C. Knowles

**Affiliations:** ^1^ Division of Biomaterials and Tissue Engineering UCL Eastman Dental Institute University College London London UK; ^2^ Department of Conservative Dentistry College of Dentistry University of Baghdad Baghdad Iraq; ^3^ School of Dentistry College of Biomedical and Lifesciences Department of Learning and Scholarship Cardiff University Cardiff UK; ^4^ School of Pharmacy Faculty of Life Sciences University College London London UK

**Keywords:** Biodegradation, biofilm resistance, cell wall

## Abstract

Root canal irrigation is an important adjunct to control microbial infection. The aim of this study was to investigate the effect of 2.5% (wt/vol) sodium hypochlorite (NaOCl) agitation on the removal, killing, and degradation of *Enterococcus faecalis* biofilm. A total of 45 root canal models were manufactured using 3D printing with each model comprising an 18 mm length simulated root canal of apical size 30 and taper 0.06. *E. faecalis* biofilms were grown on the apical 3 mm of the models for 10 days. A total of 60 s of 9 ml of 2.5% NaOCl irrigation using syringe and needle was performed, the irrigant was either left stagnant in the canal or agitated using manual (Gutta‐percha), sonic, and ultrasonic methods for 30 s. Following irrigation, the residual biofilms were observed using confocal laser scanning, scanning electron, and transmission electron microscopy. The data were analyzed using one‐way ANOVA with Dunnett *post hoc* tests at a level of significance *p* ≤ .05. Consequence of root canal irrigation indicate that the reduction in the amount of biofilm achieved with the active irrigation groups (manual, sonic, and ultrasonic) was significantly greater when compared with the passive and untreated groups (*p* < .05). Collectively, finding indicate that passive irrigation exhibited more residual biofilm on the model surface than irrigant agitated by manual or automated (sonic, ultrasonic) methods. Total biofilm degradation and nonviable cells were associated with the ultrasonic group.

## 1 Introduction

Verification has been established regarding the essential role of bacteria in the evolution of periradicular diseases (Kakehashi, Stanley, & Fitzgerald, 1965). Bacteria can adhere to surfaces and rapidly form biofilms (Costerton, Stewart, & Greenberg, 1999). A biofilm is defined as a community of microorganisms of one or more species embedded in an extracellular polymeric substance that is attached to a solid substrate (Wilson, 1996). The root canal treatment of an infected root canal system includes the microbial control through instrumentation and irrigation. Irrigation aims to lubricate the instruments, as well as remove microorganisms present in the root canal system through the chemical and flushing action (Baker, Eleazer, Averbach, & Seltzer, 1975). However, the debridement action of an irrigant within the root canal system may remain elusive when using a needle and syringe alone (Jiang, Lak, Eijsvogels, Wesselink, & Van Der Sluis, 2012). Irrigant agitation may be applied to aid the dispersal of the irrigant into the root canal system, especially into the periapical terminus of the canal (Druttman & Stock, 1989). Agitation techniques for root canal irrigant include either manual (Cunningham, Martin, & Forrest, 1982) or automated agitation (Sabins, Johnson, & Hellstein, 2003).

The topic of the efficiency of irrigation in removing bacterial biofilm has received considerable critical attention. For example, studies that include the growth of selected bacteria on a substratum surface and its subsequent exposure to the antimicrobial agent. The substrata used to grow biofilms include nitrocellulose filter membranes (Spratt, Pratten, Wilson, & Gulabivala, 2001), hydroxyapatite disks (Niazi et al., 2014), sections of root apex (Clegg, Vertucci, Walker, Belanger, & Britto, 2006), dentine disks (Stojicic, Shen, & Haapasalo, 2013), and glass (Williamson, Cardon, & Drake, 2009). However, approaches of this kind carry with them the well‐known limitation that the immersion of samples in the irrigant is different from exposure to irrigant flow within the confinement of a root canal system. Recently, there has been renewed interest in using Computational Fluid Dynamic models to measure the physical parameters associated with irrigant flow within the root canal system, however, these provide a virtual view of root canal irrigation but lack the ability to estimate the interaction between an irrigant and the biofilm (Shen et al., 2010).

Although extensive research has been carried out on irrigant biofilm interaction, the degradation and removal effect of active and passive irrigation protocols on the biofilms within the root canal system have not been closely examined. Therefore, the aim was to investigate the agitation influence of 2.5% NaOCl on the removal and degradation of *Enterococcus faecalis* biofilm.

## 2 Materials and Methods

### 2.1 Root canal model construction, biofilm generation, and irrigation experiments

The root canal models (*n* = 45) were manufactured using 3D printer in the same manner of previous study (Mohmmed et al., 2016), creating a straight canal model of 18 mm length, apical size 30, and a 0.06 taper. The models were sterilized using gas plasma with hydrogen peroxide vapor for 50 minutes.

Biofilms were grown from *Enterococcus faecalis* strain *(*ATCC 19433), which was plated onto a BHI agar (Sigma‐Aldrich, St. Louis, Montana, USA) with 5% defibrinated horse blood and incubated at 37°C in the 5% CO_2_ incubator for 24 hr. Inoculum concentration was 1.1 × 10^8^ CFU/ml, which was confirmed using six 10‐fold serial dilutions.

One ml of standard *E. faecalis* inoculum was delivered into a sterilized 7 ml plastic bijou bottle containing the sterilized half model such that the 3 mm apical portion was immersed. This was achieved using a sterile syringe and a 21‐gauge needle. The samples were then incubated at 37°C in the 5% CO_2_ incubator for 10 days. Every 2 days, half of the inoculum was discarded and replaced with fresh BHI broth (De‐Deus, Brandão, Fidel, & Fidel, 2007).

Before reassembling the two model halves, one sterile and one with a biofilm, a polyester seal film of 0.05 mm thickness was positioned on the half coated with biofilm. The two halves of the model were then held in position using four brass bolts (size 16 BA) and nuts.

The apical end of each canal was blocked using a sticky wax (Associated Dental Product Ltd, Swindon, UK). The models were divided to five groups (1–5) (*n* = 9 per group) according to the irrigation protocols. In‐group 1 (control group), the models with the biofilm were examined without irrigation. In‐group 2 (passive irrigation group), 9 ml of 2.5% NaOCl (Teepol^®^ bleach, Teepol products, Egham, UK) were delivered using a 10 ml syringe with a 27‐gauge side‐cut open‐ended needle. The needle was inserted 3 mm coronal to the canal terminus. The port opening of the needle always faced the model half containing the biofilm. The syringe was attached to a programmable precision syringe pump to deliver the irrigant in 60 s at a flow rate of 0.15 ml s^−1^, followed by 30 s of irrigant that was kept stagnant (passive) in the canal.

For group 3 (manual agitation group), irrigant was delivered for 60 s as in the group 2, then agitated for 30 s using a Gutta‐percha cone (GP) (SybronEndo, Buffalo, New York, USA). The cone with an apical ISO size 30 and .02 taper was placed 2 mm coronal to the canal terminus was used to agitate the irrigant in the root canal system with a push‐pull amplitude of approximately 3–5 mm at a frequency of 50 strokes per 30 s. A new GP cone was used with each canal model.

In group 4 (sonic agitation group), irrigant was delivered as in group 3 but agitated using EndoActivator^®^ device (Dentsply Tulsa Dental Specialties, Tulsa, OK, USA). the agitation was carried out using an EndoActivator^®^ device by placing the polymer tip with size 25 and .04 taper at 2 mm from the canal terminus, and then the agitation was continued for 30 s with high power setting. Once again, a new tip was used with each canal model.

In the ultrasonic agitation group, irrigant was delivered as in previous group but agitated using Satelec^®^ P5 ultrasonic device (Satelec, Acteon, Equipment, Merignac, France). This was carried out by placing a stainless steel instrument size and taper 20/02 (IrriSafe; Satelec Acteon, Merignac, France) of Satelec^®^ P5 Newtron piezon unit at 2 mm from the canal terminus, then the agitation was continued for 30 s. The file was energized at power setting 7 as recommended by the manufacturer. A new instrument was used with each canal model.

Following irrigation protocols, the residual NaOCl on the model surface was immediately neutralized by immersing the models in 2 ml of 5% sodium thiosulfate solution (Sigma‐Aldrich Co Ltd., Gillingham, UK) for 5 min. This reduces the active ingredient of NaOCl (hypochlorite), which becomes oxidized to sulfate (Hegde, Bashetty, & Krishnakumar, 2012).

The models in each group were then randomly divided in to three subgroups for investigation with CLSM, SEM, and TEM microscopy techniques (*n* = 3 per subgroup).

### 2.2 Preparation of the samples for confocal laser scanning microscopy

**Figure 1:**
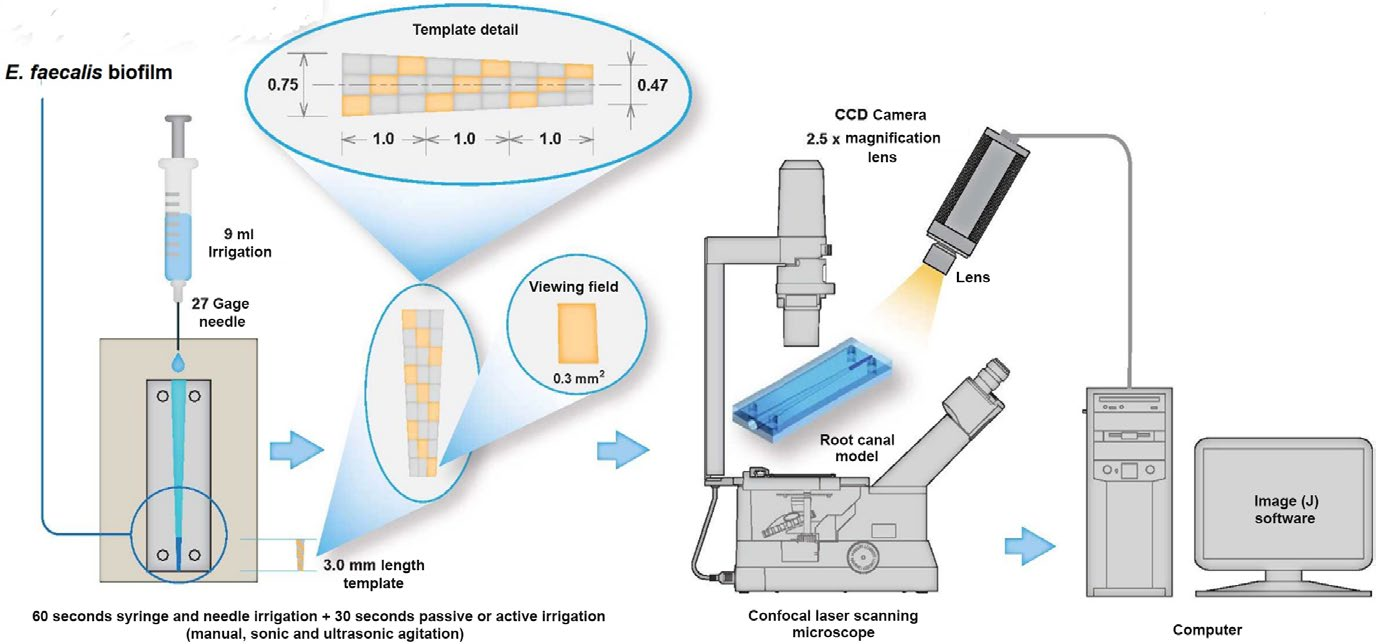
Image illustrates the set‐up of the equipment

Three models from each group were examined to assess the viability of bacterial cells in the residual surface biofilm using the Live/Dead^®^ viability stain (LIVE/DEAD BacLight; Invitrogen, Paisley, UK) and CLSM (BioRad Radiance2100, Zeiss, Welwyn Garden City, Herts, UK) along with its designated software for documentation of results. The stain was prepared by mixing 3 μl each of Syto 9 and propidium iodide compounds. The models were removed from the incubator and the stain mixture was pipetted directly onto the surface of each sample. The samples were then placed in a sealed dark box and left to incubate for 15 min at room temperature (Defives, Guyard, Oularé, Mary, & Hornez, 1999). Each sample was then placed onto the microscope stage of the CLSM and imaged with an ×20 lens using both a fluorescent and laser light source. The canal surface was imaged at 3, 2, and 1 mm from the canal terminus with the green channel indicating live cells and the red channel showing the dead bacteria. For imaging, the pixel definition was set at 1024 × 1024 pixels with no digital zoom. The representative portion was scanned at ×1 digital zoom in a simple x y two dimensional plane. The images were then constructed and manipulated using ImageJ^®^ software. For each area (1 mm^2^) of the 3 mm from the canal terminus, the sample was tested to obtain representative images of the live/dead cells by viewing three fields of 0.3 mm^2^ from within the root canal. The fields were located in the top, middle, and bottom of the tested area (Figure 1).

### 2.3 Preparation of the samples for scanning electron microscopy

Three models from each group were examined to assess the effect of 2.5% NaOCl irrigant on the residual surface biofilm using SEM. Immediately after irrigation, the models were fixed in 3% glutaraldehyde in 0.1 mol/L sodium cacodylate buffer (pH 7.4) at 4°C overnight. Then, they were dehydrated in a graded series of alcohol (50, 70, 90, and 100%), placed in hexamethyldisilazane for 5 min, and air‐dried. Samples were mounted onto aluminum pin stubs, and sputter coated with gold/palladium before examination using SEM (FEI XL30 FEG SEM, FEI, Eindhoven, Netherlands) at 5 kV. The residual biofilm on the canal surface was imaged at 3, 2, and 1 mm from the canal terminus using ×2,000 and ×8,000 magnification.

### 2.4 Preparation of the samples for transmission electron microscopy

Three models from each group were examined using TEM to further assess the effect of 2.5% NaOCl on the residual biofilm and individual cells. Following fixation in 3% glutaraldehyde in 0.1 mol/L cacodylate buffer, samples were dehydrated in a graded series of alcohol (50%, 70% and 3 × 90% for 10 min each). They were then infiltrated with LR White resin by immersion in LR White resin and 90% alcohol (ratio of 1:1) for 2 hr at 4°C, followed by a change to pure fresh LR White for 30 min, another change to fresh LR White overnight at 4°C. The following morning, the models were embedded in foil tins containing 20 ml of LR White and 30 μl LR White accelerator at room temperature. Air was excluded from the setting process by placing a piece of para‐film cut to size over the surface of the exposed resin mix in the foil tin. The resin mixture was stored overnight in the freezer for polymerization and then removed and left to warm up to room temperature.

Semithin sections of the canal (80–90) nm were cut with a Diatome diamond knife on an ultramicrotome and collected on gold 200 mesh grids. The models were then stained on the grid with 0.4% (w/v) uranyl acetate in absolute alcohol for 5 min, models were examined on a TEM (Philips CM12, FEI, Eindhoven, Netherlands) operating at 80 kV.

### 2.5 Data analyses

The mean and standard deviation values of the surface area (μ^2^) of *E. faecalis* biofilm on the canal surface by the experimental group (level from the canal terminus) were calculated by SPSS (BM Corp.Released 2013. IBM SPSS Statistics for Windows, Version 22.0.Armonk, New York, USA). The data were analyzed using one‐way analysis of variance (ANOVA), followed by Dunnett *post hoc* comparisons. A significance level of 0.05 was used throughout.

## 3 Results

### 3.1 Statistical analysis

**Figure 2:**
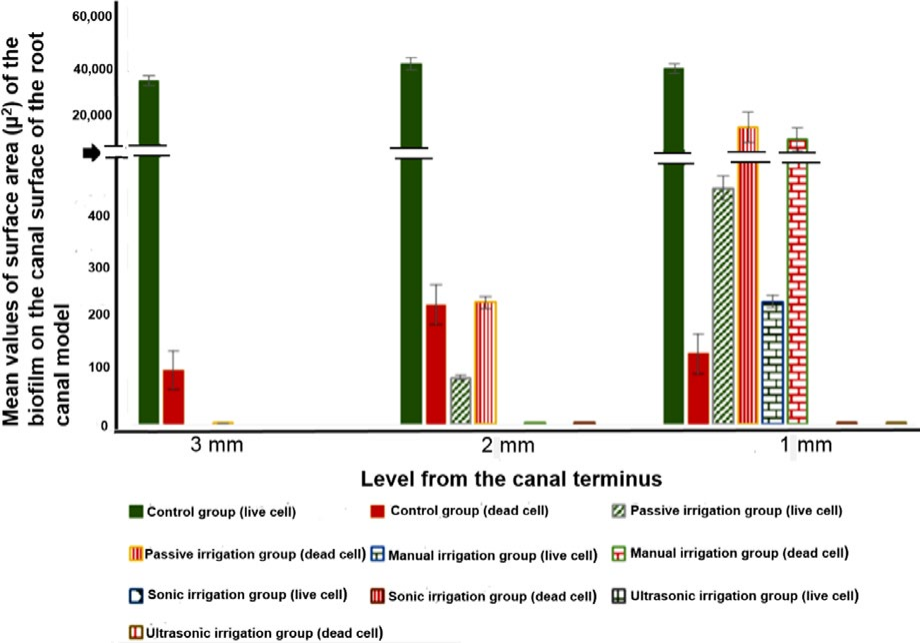
Mean values of surface area (μ^2^) of *E. faecalis* biofilm on the canal surface at 3, 2, and 1 mm from the canal terminus, before and after irrigation protocols. The black arrow on the *y*‐axis indicates breaks of different value axis scaling. Error bars are standard deviation (*n* = 3 per group)

The mean surface area values of *E. faecalis* biofilm on the root canal surface without irrigation and after 90 s passive or active irrigation protocol using 2.5% NaOCl are presented in Figure 2.

The ANOVA test revealed that the reduction in the amount of biofilm achieved with the active irrigation group groups (manual, sonic, and ultrasonic) was significantly greater when compared with the passive and untreated group (*p* < .05). Interestingly, no significant differences was found between the passive irrigation and untreated groups (*p* = .8).

For the active irrigation groups, the reduction in the amount of biofilm in the ultrasonic group was significantly [12867.3 μ^2^ (±5)], more than that in the manual group (*p* = .001), whilst it was interestingly not significantly [0.23 μ^2^ (±5)] more than that in the sonic group (*p* = .9). The reduction in the amount of biofilm in the sonic group was significantly [12867.5 (±5)] more than that in the manual group (*p* = .001).

### 3.2 Microscopic images analysis

**Figure 3:**
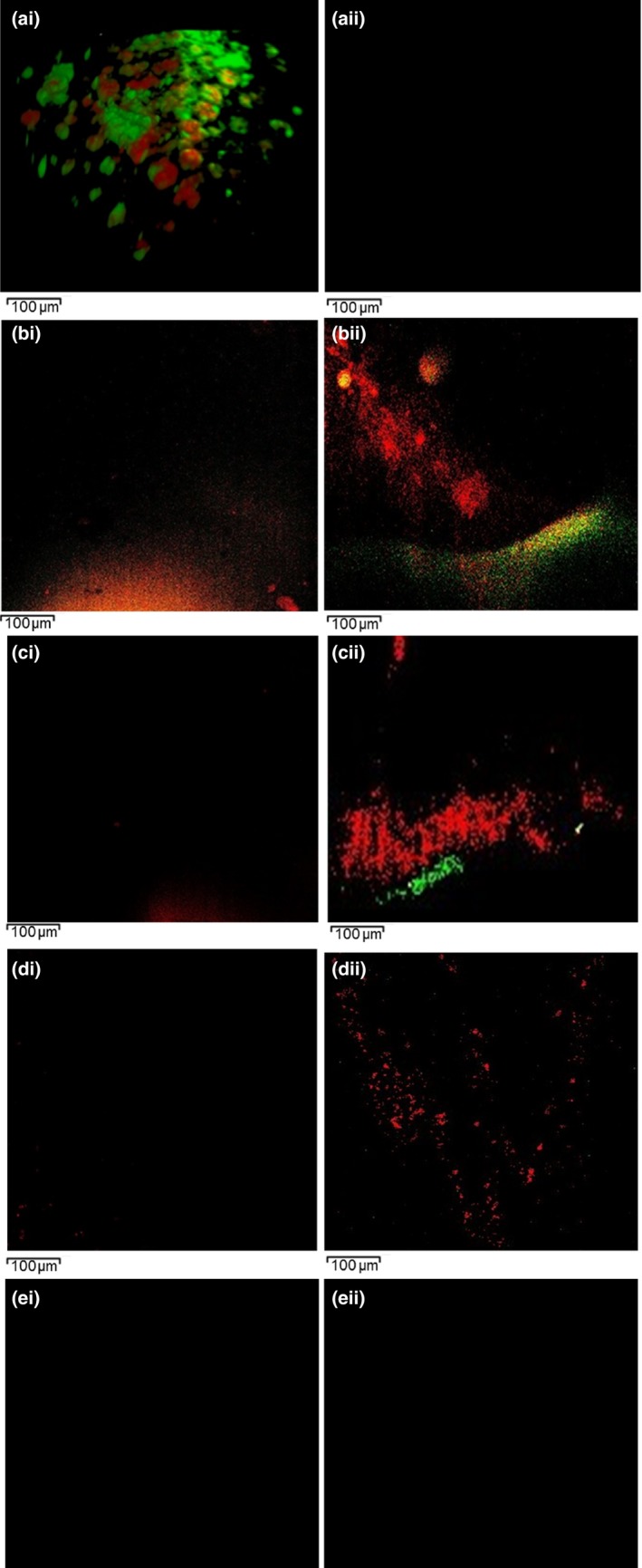
CLSM (×20 magnification) images (0.3 mm^2^) from within the root canal to illustrate (a) *E. faecalis* biofilm grown for 10 days and stained using Live/Dead^®^ viability stain with the green color indicating live cells and the red color showing the dead bacteria (control). (ai) residual biofilm at 3 mm from the canal terminus after syringe irrigation protocol. (b) Passive irrigation group; (i) residual biofilm at 2 mm from the canal terminus; (ii) residual biofilm at 1 mm from the canal terminus. (c) manual‐agitation group; (i) residual biofilm at 2 mm from the canal terminus; (ii) residual biofilm at 1 mm from the canal terminus. (d) Sonic agitation group; (i) residual biofilm at 2 mm from the canal terminus; (ii) residual biofilm at 1 mm from the canal terminus. (e) Ultrasonic agitation group; (i) residual biofilm at 2 mm from the canal terminus; (ii) residual biofilm at 1 mm from the canal terminus

The CLSM (×20 magnification) images of the biofilm on the surface of the root canal models before and after irrigation are presented in Figure 3.

In the untreated model (control group), observations of the CLSM images of the biofilm (Figure 3a) demonstrated more live cells (green) than dead cells (red). The dark background of these images indicates the nonfluorescent property of the of the model materials.

In the treated groups, the CLSM images exhibited no residual biofilm at 3 mm level from the canal terminus in all groups (Figure 3ai). At 2 mm level, the images showed no viable cells in all groups. However, dispersed clusters of residual dead biofilm (red) were more abundant in the passive irrigation group (Figure 3bi) than manual agitation group (Figure 3ci). Complete removal of biofilm was associated with the automated groups (sonic, ultrasonic) (Figure 3di & ei, respectively).

At 1 mm, the images demonstrated both viable and dead cells in the passive irrigation group (Figure 3bii) and manual (Figure 3cii) groups with greater live cells than dead cells in the former group. Regarding the automated groups, it was notable that no viable cells were detected. Moreover, the scanty clusters of the residual dead cells in the sonic (Figure 3dii) group were more than that of the ultrasonic group (Figure 3eii).

**Figure 4:**
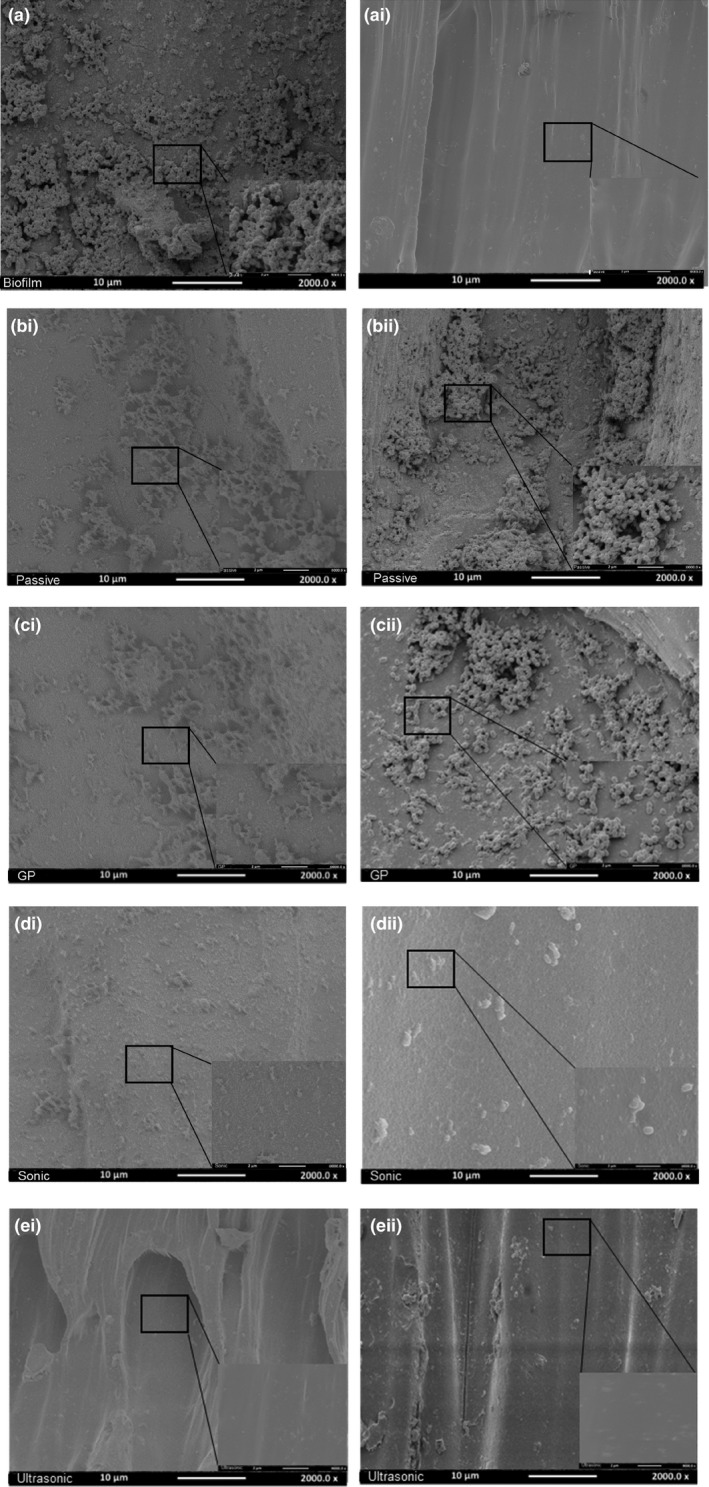
SEM images (×2,000, ×8,000 magnification) illustrate (a) *E. faecalis* biofilm grown for 10 days onto the surface of the root canal model (control). (ai) residual biofilm at 3 mm from the canal terminus after syringe irrigation protocol. (b) Passive irrigation group; (i) residual biofilm at 2 mm from the canal terminus; (ii) residual biofilm at 1 mm from the canal terminus. (c) manual‐agitation group; (i) residual biofilm at 2 mm from the canal terminus; (ii) residual biofilm at 1 mm from the canal terminus. (d) Sonic agitation group; (i) residual biofilm at 2 mm from the canal terminus; (ii) residual biofilm at 1 mm from the canal terminus. (e) Ultrasonic agitation group; (i) residual biofilm at 2 mm from the canal terminus; (ii) residual biofilm at 1 mm from the canal terminus

SEM (×2,000, ×8,000 magnification) images of the biofilm on the surface of the root canal models before and after irrigation are presented in Figure 4.

SEM assessment of the untreated biofilm (Figure 4a) illustrated typical biofilm growth with many small and larger colonies often embedded within a layer of extracellular polymeric substance.

After 2.5% NaOCl irrigation, SEM images exhibited no residual biofilm was detected at 3 mm level of all groups (Figure 4ai). SEM images of the biofilm at 2 mm showed that the least an extracellular polymeric substance (EPS) destruction and cell degradation was associated with the passive irrigation group (Figure 4bi) followed by manual (Figure 4ci), sonic (Figure 4di), and ultrasonic (Figure 4ei) groups, respectively. At 1 mm, SEM images illustrated that the biofilm appeared intact with the least bacterial cell degradation and deformation in the passive irrigation group (Figure 4bii), followed by manual (Figure 4cii), sonic (Figure 4dii) groups, respectively. Interestingly, complete biofilm removal and cell degradation were associated with the ultrasonic group.

**Figure 5:**
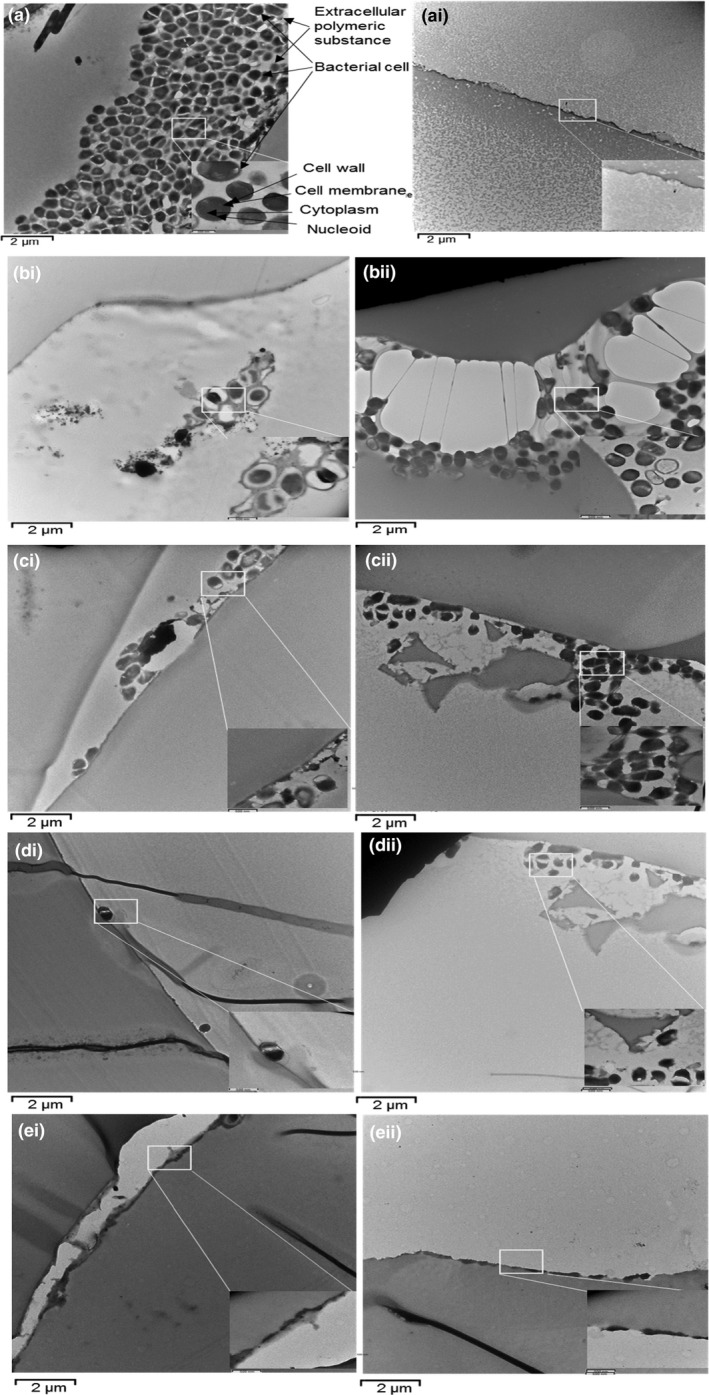
TEM (×7,100, 31,000) images illustrate (a) *E. faecalis* biofilm grown for 10 days onto the surface of the root canal model (control). (ai) residual biofilm at 3 mm from the canal terminus after syringe irrigation protocol. (b) Passive irrigation group; (i) residual biofilm at 2 mm from the canal terminus; (ii) residual biofilm at 1 mm from the canal terminus. (c) manual‐agitation group; (i) residual biofilm at 2 mm from the canal terminus; (ii) residual biofilm at 1 mm from the canal terminus. (d) Sonic agitation group; (i) residual biofilm at 2 mm from the canal terminus; (ii) residual biofilm at 1 mm from the canal terminus. (e) Ultrasonic agitation group; (i) residual biofilm at 2 mm from the canal terminus; (ii) residual biofilm at 1 mm from the canal terminus

The TEM (×7,100, ×31,000) images of the biofilm on the surface of the root canal models before and after irrigation using passive irrigation, manual, and automated agitation protocols are presented in Figure 5.

TEM assessment of the untreated biofilm on the root canal model (Figure 5a) showed that it consisted of bacterial cells surrounded by EPS. At higher magnification, the bacterial cells exhibited a distinct coccoid appearance, a smooth and intact outer cell wall, a cell membrane surrounding the cytoplasm, and electron‐dense irregularly shaped areas within the cell,

After 2.5% NaOCl irrigation, TEM images exhibited no residual biofilm was detected at 3 mm level of all groups (Figure 5ai). The TEM images of the residual biofilm at 2 mm demonstrated extensive biofilm degradation, bacterial cell deformations/perforations, and apparent removal of EPS in passive irrigation (Figure 5bi) and manual (Figure 5ci) groups. In comparison, complete biofilm degradation, removal, and cell damage were associated with Sonic (Figure 5di) and ultrasonic (Figure 5ei) groups. At 1 mm, bacterial cells in the residual biofilm seemed to maintain their cell wall and structural integrity in both passive irrigation (Figure 5bii) and manual (Figure 5cii) groups. In comparison, damaged cells of the residual biofilm were abundant in the sonic (Figure 5dii) group. Whilst, complete biofilm disintegration were associated with the ultrasonic (Figure 5eii) groups.

Generally, passive irrigation with NaOCl resulted in more residual biofilm than NaOCl agitated by manual or automated (sonic, ultrasonic) method. Total biofilm degradation and nonviable cells were associated with automated groups.

## 4 Discussion

The experiments were successful in testing the aim, which was to determine the effect of different irrigation protocols on the ability of 2.5% NaOCl irrigant to remove and degrade a single species biofilm within a simulated root canal model. A NaOCl irrigant (2.5%) was selected for the irrigation procedure since it constitutes the most frequently used irrigant in root canal treatment (Baumgartner & Cuenin, 1992).

The findings indicated that the type of irrigation protocol used could be crucial to achieve complete loss of cell viability (killing), degradation, and removal of the bacterial biofilm. Overall, passive irrigation was ineffective, whilst ultrasonic agitation of 2.5% NaOCl seemed the most effective followed by sonic and manual agitation protocols. The results of the data analysis of the biofilm on the root canal surface were confirmed by microscopic image evaluation. Analysis of the microscopic images (CLSM, SEM, and TEM) of the 1 mm^2^ surface area of the root canals at 3 mm showed no marked differences in the biofilm layer, in terms of killing, cell wall destruction and complete removal of biofilm. A possible explanation for these results may be related to fluid dynamics around the tip of the side cut needle, that creates an eddy with a diameter of approximately 1 mm in the area around to the needle tip (Verhaagen, Boutsioukis, Heijnen, Van Der Sluis, & Versluis, 2012), as well as, the chemical action, which related to the oxidizing effect of the OCl^−^/HOCl^−^ of the NaOCl (Boutsioukis, Lambrianidis, & Kastrinakis, 2009).

A marked difference was found between the passive and active irrigation protocols at 2 and 1 mm. The reduction in killing and destruction of the biofilm by NaOCl in the passive group could be related to the decrease in velocity (Verhaagen et al., 2012) and possible regions of stagnation of the irrigant (Ram, 1977). Another possible explanation for this is that air bubbles may become trapped in the apical region of the root canal system during needle and syringe irrigation (Tay et al., 2010). This suggests that it may be impossible to achieve complete removal of biofilm using passive irrigation in the apical part of the canal. In comparison, the greater biofilm degradation and cell killing in active irrigation groups may be related to the impact of agitation on the dissolving capacity of NaOCl (Moorer & Wesselink, 1982). Furthermore, agitation enhances the mixing of fresh irrigant with the stagnant, used fluid in the apical part of the canal (Bronnec, Bouillaguet, & Machtou, 2010). However, the difference in effectiveness of the techniques used to agitate NaOCl inside the root canal may be related to space restrictions of the root canal that interfere with the agitation method.

The difference between the manual agitation group and the automated groups (sonic, & ultrasonic) could be attributed to the fact that the manual push–pull motion of a gutta‐percha point generates a frequency that is less efficient than the automated methods (Layton, Wu, Selvaganapathy, Friedman, & Kishen, 2015). However, the manual agitation method is easy to practice and is not expensive. Moreover, it allowed more biofilm degradation and removal than passive irrigation (Huang, Gulabivala, & Ng, 2008).

The difference between EndoActivator sonic and ultrasonic agitation may be due to the driving frequency of the ultrasonic device, which was higher than that of the sonic device. A higher frequency produces a higher flow velocity of NaOCl irrigant (Verhaagen et al., 2012), and this may result in an increased biofilm removal by ultrasonic device.

The possible limitation of the study is that the sample size was relatively small, although statistically significant differences were indeed found. This indicates that the model is sensitive enough; such statistical significance does not tell us how big the difference is. This is important in clinical terms since it may alter the clinical approach of the irrigation procedure (Trope, Delano, & Ørstavik, 1999). A robust calculation of the optimal sample size is crucial to be considered in future work for the minimization of the risk of type I or II errors (Schuurs, Wu, Wesselink, & Duivenvoorden, 1993).

## 5 Conclusion

Within the limitations of this study, passive irrigation using 2.5% NaOCl exhibited more residual biofilm on the model surface than 2.5% NaOCl irrigant agitated by manual or automated (sonic, ultrasonic) method. Total biofilm degradation and nonviable cells were associated with ultrasonic group.

## Conflict of Interest

None declared.

## References

[mbo3455-bib-0001] Baker, N. A. , Eleazer, P. D. , Averbach, R. E. , & Seltzer, S. (1975). Scanning electron microscopic study of the efficacy of various irrigating solutions. Journal of Endodontics, 1(4), 127–135. 765422 10.1016/S0099-2399(75)80097-5

[mbo3455-bib-0002] Baumgartner, J. C. , & Cuenin, P. R. (1992). Efficacy of several concentrations of sodium hypochlorite for root canal irrigation. Journal of Endodontics, 18(12), 605–612. 1298800 10.1016/S0099-2399(06)81331-2

[mbo3455-bib-0003] Boutsioukis, C. , Lambrianidis, T. , & Kastrinakis, E. (2009). Irrigant flow within a prepared root canal using various flow rates: A Computational Fluid Dynamics study. International Endodontic Journal, 42(2), 144–155. 19134043 10.1111/j.1365-2591.2008.01503.x

[mbo3455-bib-0004] Bronnec, F. , Bouillaguet, S. , & Machtou, P. (2010). Ex vivo assessment of irrigant penetration and renewal during the final irrigation regimen. International Endodontic Journal, 43(8), 663–672. 20491986 10.1111/j.1365-2591.2010.01723.x

[mbo3455-bib-0005] Clegg, M. , Vertucci, F. , Walker, C. , Belanger, M. , & Britto, L. (2006). The effect of exposure to irrigant solutions on apical dentin biofilms in vitro. Journal of Endodontics, 32(5), 434–437. 16631843 10.1016/j.joen.2005.07.002

[mbo3455-bib-0006] Costerton, J. W. , Stewart, P. S. , & Greenberg, E. (1999). Bacterial biofilms: A common cause of persistent infections. Science, 284(5418), 1318–1322. 10334980 10.1126/science.284.5418.1318

[mbo3455-bib-0007] Cunningham, W. T. , Martin, H. , & Forrest, W. R. (1982). Evaluation of root canal debridement by the endosonic ultrasonic synergistic system. Oral Surgery, Oral Medicine, Oral Pathology, 53(4), 401–404. 10.1016/0030-4220(82)90442-x 6952152

[mbo3455-bib-0008] De‐Deus, G. , Brandão, M. , Fidel, R. , & Fidel, S. (2007). The sealing ability of GuttaFlow™ in oval‐shaped canals: An ex vivo study using a polymicrobial leakage model. International Endodontic Journal, 40(10), 794–799. 17714465 10.1111/j.1365-2591.2007.01295.x

[mbo3455-bib-0009] Defives, C. , Guyard, S. , Oularé, M. , Mary, P. , & Hornez, J. (1999). Total counts, culturable and viable, and non‐culturable microflora of a French mineral water: A case study. Journal of Applied Microbiology, 86(6), 1033–1038. 10389250 10.1046/j.1365-2672.1999.00794.x

[mbo3455-bib-0010] Druttman, A. , & Stock, C. (1989). An in vitro comparison of ultrasonic and conventional methods of irrigant replacement. International Endodontic Journal, 22(4), 174–178. 2637222 10.1111/j.1365-2591.1989.tb00920.x

[mbo3455-bib-0011] Hegde, J. , Bashetty, K. , & Krishnakumar, U. G. (2012). Quantity of sodium thiosulfate required to neutralize various concentrations of sodium hypochlorite. Asian Journal of Pharmaceutical and Health Sciences, 2(3), 390–393.

[mbo3455-bib-0012] Huang, T. Y. , Gulabivala, K. , & Ng, Y. L. (2008). A bio‐molecular film ex‐vivo model to evaluate the influence of canal dimensions and irrigation variables on the efficacy of irrigation. International Endodontic Journal, 41(1), 60–71. 17916068 10.1111/j.1365-2591.2007.01317.x

[mbo3455-bib-0013] Jiang, L.‐M. , Lak, B. , Eijsvogels, L. M. , Wesselink, P. , & Van Der Sluis, L. W. (2012). Comparison of the cleaning efficacy of different final irrigation techniques. Journal of Endodontics, 38(6), 838–841. 22595122 10.1016/j.joen.2012.03.002

[mbo3455-bib-0014] Kakehashi, S. , Stanley, H. , & Fitzgerald, R. (1965). The effects of surgical exposures of dental pulps in germ‐free and conventional laboratory rats. Oral Surgery, Oral Medicine, Oral Pathology, 20(3), 340–349. 10.1016/0030-4220(65)90166-0 14342926

[mbo3455-bib-0015] Layton, G. , Wu, W.‐I. , Selvaganapathy, P. R. , Friedman, S. , & Kishen, A. (2015). Fluid dynamics and biofilm removal generated by syringe‐delivered and 2 ultrasonic‐assisted irrigation methods: A novel experimental approach. Journal of Endodontics, 41(6), 884–889. 25749254 10.1016/j.joen.2015.01.027

[mbo3455-bib-0016] Mohmmed, S. A. , Vianna, M. E. , Penny, M. R. , Hilton, S. T. , Mordan, N. , & Knowles, J. C. (2016). A novel experimental approach to investigate the effect of different agitation methods using sodium hypochlorite as an irrigant on the rate of bacterial biofilm removal from the wall of a simulated root canal model. Dental Materials, 32(10), 1289–1300. 27515530 10.1016/j.dental.2016.07.013

[mbo3455-bib-0017] Moorer, W. , & Wesselink, P. (1982). Factors promoting the tissue dissolving capability of sodium hypochlorite. International Endodontic Journal, 15(4), 187–196. 6964523 10.1111/j.1365-2591.1982.tb01277.x

[mbo3455-bib-0018] Niazi, S. , Clark, D. , Do, T. , Gilbert, S. , Foschi, F. , Mannocci, F. , & Beighton, D. (2014). The effectiveness of enzymic irrigation in removing a nutrient‐stressed endodontic multispecies biofilm. International Endodontic Journal, 47(8), 756–768. 24246147 10.1111/iej.12214

[mbo3455-bib-0019] Ram, Z. (1977). Effectiveness of root canal irrigation. Oral Surgery, Oral Medicine, Oral Pathology, 44(2), 306–312. 10.1016/0030-4220(77)90285-7 268582

[mbo3455-bib-0020] Sabins, R. A. , Johnson, J. D. , & Hellstein, J. W. (2003). A comparison of the cleaning efficacy of short‐term sonic and ultrasonic passive irrigation after hand instrumentation in molar root canals. Journal of Endodontics, 29(10), 674–678. 14606795 10.1097/00004770-200310000-00016

[mbo3455-bib-0021] Schuurs, A. , Wu, M. K. , Wesselink, P. , & Duivenvoorden, H. (1993). Endodontic leakage studies reconsidered. Part II. Statistical aspects. International Endodontic Journal, 26(1), 44–52. 8473034 10.1111/j.1365-2591.1993.tb00541.x

[mbo3455-bib-0022] Shen, Y. , Gao, Y. , Qian, W. , Ruse, N. D. , Zhou, X. , Wu, H. , & Haapasalo, M. (2010). Three‐dimensional numeric simulation of root canal irrigant flow with different irrigation needles. Journal of Endodontics, 36(5), 884–889. 20416439 10.1016/j.joen.2009.12.010

[mbo3455-bib-0023] Spratt, D. , Pratten, J. , Wilson, M. , & Gulabivala, K. (2001). An in vitro evaluation of the antimicrobial efficacy of irrigants on biofilms of root canal isolates. International Endodontic Journal, 34(4), 300–307. 11482142 10.1046/j.1365-2591.2001.00392.x

[mbo3455-bib-0024] Stojicic, S. , Shen, Y. , & Haapasalo, M. (2013). Effect of the source of biofilm bacteria, level of biofilm maturation, and type of disinfecting agent on the susceptibility of biofilm bacteria to antibacterial agents. Journal of Endodontics, 39(4), 473–477. 23522539 10.1016/j.joen.2012.11.024

[mbo3455-bib-0025] Tay, F. R. , Gu, L.‐S. , Schoeffel, G. J. , Wimmer, C. , Susin, L. , Zhang, K. , … Pashley, D. H. (2010). Effect of vapor lock on root canal debridement by using a side‐vented needle for positive‐pressure irrigant delivery. Journal of Endodontics, 36(4), 745–750. 20307757 10.1016/j.joen.2009.11.022 PMC2844877

[mbo3455-bib-0026] Trope, M. , Delano, E. O. , & Ørstavik, D. (1999). Endodontic treatment of teeth with apical periodontitis: Single vs. multivisit treatment. Journal of Endodontics, 25(5), 345–350. 10530259 10.1016/S0099-2399(06)81169-6

[mbo3455-bib-0027] Verhaagen, B. , Boutsioukis, C. , Heijnen, G. , Van Der Sluis, L. , & Versluis, M. (2012). Role of the confinement of a root canal on jet impingement during endodontic irrigation. Experiments in Fluids, 53(6), 1841–1853.

[mbo3455-bib-0028] Williamson, A. E. , Cardon, J. W. , & Drake, D. R. (2009). Antimicrobial Susceptibility of Monoculture Biofilms of a Clinical Isolate of *Enterococcus faecalis* . Journal of Endodontics, 35(1), 95–97. 19084133 10.1016/j.joen.2008.09.004

[mbo3455-bib-0029] Wilson, M. (1996). Susceptibility of oral bacterial biofilms to antimicrobial agents. Journal of Medical Microbiology, 44(2), 79–87. 8642580 10.1099/00222615-44-2-79

